# Cognition, serum BDNF levels, and BDNF Val66Met polymorphism in type 2 diabetes patients and healthy controls

**DOI:** 10.18632/oncotarget.23342

**Published:** 2017-12-16

**Authors:** Yan-Feng Zhen, Xing-Yu Liu, Dong-Hao Zhou, Xiangdong Du, Guangzhong Yin, Yingyang Zhang, Hui Fang, Gang Xu, Jair C. Soares, Xiang Yang Zhang

**Affiliations:** ^1^ Department of Endocrinology, Tangshan Gongren Hospital, Tangshan, China; ^2^ Department of Neurosurgery, Tangshan Gongren Hospital, Tangshan, China; ^3^ Department of Endocrinology, Linyi People’s Hospital, Linyi, China; ^4^ Suzhou Psychiatric Hospital, The Affiliated Guangji Hospital of Soochow University, Suzhou, China; ^5^ Department of Burn, Tangshan Gongren Hospital, Tangshan, China; ^6^ Department of Psychiatry and Behavioral Sciences, The University of Texas Health Science Center at Houston, Houston, TX, USA

**Keywords:** type 2 diabetes, brain-derived neurotrophic factor, cognition, polymorphism, association

## Abstract

**Background and aims:**

Type 2 diabetes (T2DM) is associated with cognitive deficits. However, their pathophysiological mechanisms are still unknown. Recent study suggests that brain-derived neurotrophic factor (BDNF) is correlated with cognitive deficits in T2DM patients. This study was to determine whether altered serum BDNF levels and cognitive deficits depended on the *BDNF* Val66Met polymorphism in T2DM.

**Results:**

The *BDNF* Val66Met polymorphism may not contribute directly to the susceptibility to T2DM. The total and nearly all index scores (all *p* < 0.01) except for the attention and visuospatial/constructional indexes (both *p* > 0.05) of RBANS were markedly decreased in T2DM compared with healthy controls. Serum BDNF levels were significantly lower in patients than that in controls (*p* < 0.001), and BDNF was positively associated with delayed memory in patients (*p* < 0.05). The Met variant was associated with worse delayed memory performance among T2DM patients but not among normal controls. Moreover, serum BDNF was positively associated with delayed memory among Met homozygote patients (*β* = 0.29, *t* = 2.21, *p* = 0.033), while serum BDNF was negatively associated the RBANS total score (*β* = –0.92, *t* = –3.40, *p* = 0.002) and language index (*β* = −1.17, *t* = –3.54, *p* = 0.001) among Val homozygote T2DM patients.

**Conclusions:**

*BDNF* gene Val66Met variation may be associated with cognitive deficits in T2DM, especially with delayed memory. The association between lower BDNF serum levels and cognitive impairment in T2DM is dependent on the *BDNF* Val66Met polymorphism.

**Methods:**

We recruited 311 T2DM patients and 346 healthy controls and compared them on the Repeatable Battery for the Assessment of Neuropsychological Status (RBANS), serum BDNF levels, and the *BDNF* Val66Met polymorphism.

## INTRODUCTION

Type 2 diabetes (T2DM) is often accompanied with cognitive dysfunction, and has an increased rate for developing dementia [1] and Alzheimer’s disease (AD) [2–4]. Previous studies have reported that T2DM was associated with cognitive deficits in certain domains, including immediate and delayed memory, processing speed, learning, as well as executive function [5–7]. Yet, the exact mechanisms underlying these decrements of cognitive function in T2DM are still unclear.

Brain-derived neurotrophic factor (BDNF) is a member of the neurotrophin family that modulates synaptic transmission [8] and hippocampal neuroplasticity that is correlated with learning and memory [9]. More specifically, BDNF induces long-term potentiation (LTP), which is thought to underlie learning and memory [10]. In BDNF knockout mice, hippocampal LTP is significantly decreased, but reversed by administrating exogenous BDNF or increasing BDNF expression [8]. Furthermore, using gene knockout or antisense RNA to inhibit the production of endogenous BDNF resulted in impairment in spatial learning and memory [11], suggesting the critical role of BDNF in learning and memory in the hippocampus [12]. Indeed, previous studies showed that a functional polymorphism Val66Met in the ‘pro’ region of *BDNF* was associated with a reduction in hippocampal volume [13] and hippocampal-dependent memory in healthy human subjects [9, 12, 13]. Moreover, some studies found that this polymorphism could influence vulnerability of the brain structural network [14] and human memory-related hippocampal activity [15] in non-diabetic populations. For example, a previous study showed that individuals with Met alleles displayed episodic memory deficiencies [9]. A subsequent study also showed that the Met allele correlated with poor medial temporal lobe-related memory performance [16]. Our study also demonstrated a correlation between the Met allele and cognitive deficit in visuospatial/ constructional index in both schizophrenia subjects and healthy controls [17]. Interestingly, Met homozygotes had volume deficits in gray matter, such as frontal, temporal, and thalamus areas in cognitive-declined diseases such as AD and depression [18–20]. Val-allele carriers showed better integrities of fiber tracts in white matter compared to Met homozygotes in the occipital area as well as in frontal temporal lobe [21]. Therefore, we hypothesized that *BDNF* Met-allele carriers would present poorer cognition compared to Val homozygotes in patients with T2DM. However, to the best of our knowledge, the association between Val66Met polymorphism of *BDNF* and the cognitive performance in T2DM has not been investigated.

To date, several studies have showed the alterations of circulating BDNF in T2DM patients, with mixed results. For example, some studies found decreased BDNF levels in T2DM [22–25]; however, the opposite results were also reported in newly diagnosed T2DM patients [26, 27]. The inconsistent results could be attributed to several factors, such as sampling of patients with different complications, different illness courses and clinical profiles, exposure for different medications, or the biological heterogeneity [9, 22]. Hence, the peripheral levels of BDNF merit further investigation in T2DM patients.

Previous research indicates that serum BDNF levels are positively associated with memory and general cognitive performance in healthy seniors [28]. This is also supported by a significantly lower level of BDNF in individuals with cognitive decline-associated disorders, such as AD and mild cognitive impairment [29]. Our previous study found both cognitive impairment and decreased BDNF serum levels in T2DM patients, as well as a positive correlation between delayed memory and BDNF levels in these patients [30]. Interestingly, our finding was confirmed by a subsequent study showing significant association between cognitive impairment and low BDNF levels in T2DM patients [31], suggesting that BDNF plays an important role in cognitive impairment in T2DM patients.

However, none have explored the inter-relationships among cognitive function, BDNF serum levels and *BDNF* genotype in T2DM patients versus healthy controls. Thus, the purposes of this study were to explore the inter-relationships among cognitive function, BDNF serum levels and *BDNF* Val66Met gene polymorphism in T2DM as well as healthy subjects. Our main aims were: (1) to confirm the association between peripheral BDNF levels and cognitive performance in both T2DM and healthy subjects; (2) to examine whether cognitive performance may be different in the *BDNF* genotype subgroups; and (3) to investigate whether the *BDNF* genotype impacts the relationship of cognitive performance and BDNF serum levels.

## RESULTS

Table 1 shows the clinical and demographic data. The patients’ average age was 54.9 ± 10.7 years, and their illness duration was 6.0 ± 1.0 years. BMI, serum TC, fasting glucose and TG were all greater in T2DM patients than those in the healthy subjects (all *p* < 0.01).

**Table 1:** Demographic characteristics and BDNF allele and genotype distributions in the controls and patients with type 2 diabetes

	Type 2 diabetes patients (*n* = 311)	Normal controls (*n* = 346)	Statistics (*p* value)
Sex (M/F)	136/175	138/208	0.99 (0.32)
Age (years)	54.93 ± 10.73	53.43 ± 9.86	3.5 (0.06)
Education (years)	9.82 ± 3.49	10.01 ± 5.85	0.21 (0.65)
BMI(kg/m^2^)	26.02 ± 3.73	24.86 ± 4.60	12.0 (<0.01)
WHR	0.92 ± 0.25	0.87 ± 0.07	8.6 (<0.01)
Fasting glucose (mmmol/l)	8.76 ± 2.96	5.02 ± 1.42	405.7 (<0.01)
Lipids (mmmol/l)			
Total cholesterol	5.77 ± 2.02	4.94 ± 1.16	38.7 (<0.01)
Triglyceride	4.13 ± 4.92	1.38 ± 0.96	97.0 (<0.01)
Duration of illness (years)	6.00 ± 1.10	NA	
HbA_1_c (%)(mmol/mol)	7.42 ± 2.02	NA	
Serum BDNF level (ng/ml)	7.73 ± 2.95	11.56 ± 2.67	295.2 (<0.01)
Allele frequency			
Val	51.0%	51.9%	0.11 (0.74)
Met	49.0%	48.1%	
Genotype frequency			
Val/Val	76 (24.4%)	88 (25.4 %)	0.12 (0.94)
Val/Met	165 (53.1%)	183 (52.9%)	
Met/ Met	70 (22.5%)	75 (21.7%)	

Abbreviation: *BMI* body mass index, *WHR* Waist-to-hip ratio, *HbA1c* Hemoglobin A1_C_.

Both the patients and healthy controls’ genotype distributions were in HWE (both *p* > 0.05). No significant differences in the *BDNF* genotype and allele frequencies were observed between the patient and control subjects (*Χ*^*2*^ = 0.12, *df* = 2, *p* > 0.05 and *Χ*^*2*^ = 0.11, *df* = 1, *p* > 0.05, respectively) (Table 1). In addition, there was no significant differences in genotype distributions between obese and non-obese groups (*p* > 0.05).

### BDNF serum levels in the patient and control subjects

BDNF levels were significantly decreased in T2DM patients than that in healthy subjects (7.7 ± 3.0 vs. 11.6 ± 2.7 ng/ml, *F* = 295.2, *df* = 1, 638, *p* < 0.001) (Table 1). After controlling for gender, age, education, BMI, fasting glucose, TC and TG, BDNF levels were still lower in patients than that in healthy subjects (*F* = 29.1, *df* = 8, 537, *p* < 0.001).

Further, BDNF levels were negatively associated with HbA_1_c (*r* = –0.13, *df* = 264, *p* = 0.029) in T2DM patients, and inversely with age (*r* = –0.13, *df* = 332, *p* < 0.05) in controls. In addition, there was no significant relationship between BDNF levels and any other clinical variables in either T2DM patients or controls (all *p* > 0.05).

### Cognitive function in the patient and control subjects

Cognitive test by the RBANS were available for 311 patients and 346 healthy controls (Table 2). RBANS total score and its three domain indexes, immediate memory, language, delayed memory were significantly lower in patients than in healthy controls (all *p* < 0.001). These significant differences still existed after controlling for gender, age, education, BMI, fasting glucose, TC and TG in the ANOVA as covariates (Table 2). Also, these significant differences passed Bonferroni corrections.

**Table 2:** Total and index scores on the RBANS in patients with type 2 diabetes and normal controls

	Type 2 diabetes (*n* = 311)	Controls (*n* = 346)	*F*	*P*	Adjusted *F*	*P*
Immediate memory	69.45 ± 14.46	76.70 ± 17.64	32.7	<0.001	11.8	<0.01
Visuospatial/constructional	81.83 ± 16.06	81.98 ± 15.41	0.02	>0.05	0.01	>0.05
Language	89.05 ± 13.50	94.25 ± 13.03	25.2	<0.001	14.2	<0.001
Attention	85.74 ± 16.73	88.17 ± 20.26	2.8	>0.05	0.08	>0.05
Delayed memory	78.56 ± 13.56	87.84 ± 14.72	70.0	<0.001	17.5	<0.001
Total	75.50 ± 13.42	81.45 ± 15.00	28.4	<0.001	9.4	<0.01

Note: Adjusted *F* shows the *F* value controlled for sex, age, education, BMI, TC, TG and fasting glucose.

### Association of BDNF with cognitive performance

Serum BDNF levels were positively correlated with the RBANS total score (*r* = 0.13, *df* = 306, *p* < 0.05) and delayed memory index score (*r* = 0.22, *df* = 306, *p* < 0.001) in T2DM patients. However, there was no significant correlation of BDNF with any index or RBANS total scores in the controls (all *p* > 0.05).

For the controls, multiple regression analysis showed that BDNF did not contribute to any RBANS test scores (all *p* > 0.05). For the patients, multiple regression analysis demonstrated that age (*β* = −0.23, *t* = −3.71, *p* < 0.001), education (*β* = 0.48, *t* = 8.04, *p* < 0.001), and BDNF (*β* = 0.12, *t* = 2.04, *p* = 0.043) contributed to the RBANS total score. Furthermore, education (*β* = 0.30, *t* = 4.55, *p* < 0.001) and BDNF (*β* = 0.21, *t* = 3.26, *p* < 0.01) were associated with the RBANS delayed memory.

### Effects of BDNF genotype on serum in patients and controls

Table 3 showed that the T2DM patients had significantly lower BDNF levels than healthy subjects when sub-grouped by *BDNF* genotype (all *p* < 0.01). However, BDNF levels were not significantly different among the three *BDNF* genotype subgroups in either patients or healthy subjects (both *p* > 0.05), without genotype × diagnosis effect (*p* > 0.05), suggesting that the *BDNF* genotype does not adjust the BDNF levels in both the patient and healthy subjects. After controlling for gender, age, education, BMI, fasting glucose, TC and TG, we still did not observe significant differences in BDNF levels among the three genotypic subgroups in these two groups (both *p* > 0.05).

**Table 3:** Serum BDNF levels (ng/ml) in controls and type 2 diabetes by genotype groupings

	Val/Val	Met/Val	Met/Met
Controls (*n* = 332)	11.5 ± 2.4	11.5 ± 2.9	11.8 ± 2.4
Type 2 diabetes (*n* = 306)	8.2 ± 3.0	7.6 ± 3.0	7.5 ± 2.9
*p* value^a^	<0.01	<0.01	<0.01

^a^Indicates the comparison between patients and controls by genotype groupings. There was no significant effect of genotype on serum BDNF levels (*p* > 0.05) or genotype × diagnosis effect (*p* > 0.05).

### Effects of BDNF genotypes on cognitive performance in patients and controls

Cognitive test by RBANS were performed in 311 patients and 346 healthy controls. Table 4 showed their RBANS total and index scores, and the effects of the BDNF Val66Met polymorphism on the RBANS total and index scores.

**Table 4:** Comparisons of total and index scores on the RBANS by diagnostic and genotype groupings

Cognitive index	Type 2 diabetes			Controls			Genotype, *F* (*p* value)	Genotype× diagnosis, *F* (*p* value)
	Val/Val (*n* = 76)	Met/Val (*n* = 165)	Met/Met (*n* = 70)	Val/Val (*n* = 88)	Met/Val (*n* = 183)	Met/Met (*n* = 75)		
Immediate memory	72.2 ± 14.2	68.9 ± 14.4	67.9 ± 14.9	79.2 ± 16.9	75.3 ± 18.2	77.1 ± 17.1	2.9 (0.06)	0.4 (0.7)
Visuospatial/ constructional	80.5 ± 16.3	82.4 ± 16.1	82.0 ± 15.9	82.1 ± 14.9	81.3 ± 15.4	83.5 ± 16.1	0.3 (0.7)	0.6 (0.6)
Language	91.2 ± 13.0	88.5 ± 12.8	88.0 ± 15.4	95.8 ± 12.2	94.3 ± 13.7	92.3 ± 12.3	2.6 (0.07)	0.2 (0.8)
Attention	87.5 ± 15.4	85.9 ± 17.3	83.5 ± 16.8	91.1 ± 18.6	87.2 ± 21.0	87.1 ± 20.2	2.1 (0.13)	0.3 (0.7)
Delayed memory	82.0 ± 14.1	77.7 ± 13.1	76.8 ± 13.5	89.4 ± 14.5	87.5 ± 15.0	86.8 ± 14.5	**3.4 (0.04)** ^a^	0.5 (0.6)
Total	77.5 ± 12.7	75.3 ± 13.2	73.9 ± 14.5	83.4 ± 13.4	80.7 ± 15.5	80.9 ± 15.5	2.2 (0.11)	0.2 (0.9)

^a^There were significant genotype effects on the delayed memory index score (*F* = 3.4, *p* < 0.05). In addition, there was also a linear negative correlation between the number of Met 66 alleles and the language, immediate and delayed memory indexes and RBANS total score in patients (all *p* < 0.05). There was no significant effect of genotype×diagnosis on RBANS total score and all the five subtest indexes (all *p* > 0.05).

As shown in Table 4, the delayed memory score was significantly different among the three genotypes (*F* = 3.4, *p* = 0.03) in T2DM patients. The Met/Met group displayed significantly lower delayed memory score than Va/Val group (*p* < 0.05). However, no significant difference was observed in the RBANS total and index scores among the three genotype groups in the controls (all *p* > 0.05) (data not shown).

Further, there was a linear negative association of the number of Met 66 alleles (dummy number, 0, 1, or 2) with the language (*β* = –0.14, *t* = –2.23, *p* < 0.05), immediate (*β* = –0.26, *t* = −4.17, *p* < 0.001) and delayed memory (*β* = –0.26, *t* = –4.01, *p* < 0.001) and the RBANS total scores (*β* = –0.17, *t* = –2.90, *p* < 0.01) in the T2DM patients.

### BDNF genotypic effects on associations of BDNF serum levels with cognitive functioning

Regression analyses found a significantly positive association between BDNF levels and delayed memory score (*β* = 0.29, *t* = 2.21, *p* = 0.033) in Met homozygote patients, as well as negative associations between BDNF and RBANS total score (*β* = –0.92, *t* = –3.40, *p* = 0.002) and language index (*β* = –1.17, *t* = –3.54, *p* = 0.001). However, among Val/Met heterozygous patients, no significant associations were found between the BDNF levels and any RBANS scores (all *p* > 0.05).

## DISCUSSION

In our current study, there was no significant difference in the *BDNF* Val66Met polymorphism between T2DM and the controls, which is consistent with a study in a Caucasian population in Denmark [22] and in a Chinese population [24], suggesting that the *BDNF* Val66Met polymorphism may be not associated with susceptibility to T2DM directly. Interestingly, the *BDNF* Val66Met polymorphism has been studied for possible association with obesity, a key pathogenic factor in development of T2DM, but reports are contradictory [32–35]. In our present, we did not find correlation of the *BDNF* Val66Met polymorphism with obesity in a Chinese Han population. One possible reason for this inconsistent result may be associated with different ethnicities with variable allele frequency distribution of *BDNF* Val66Met. For example, in our study, the frequency of the Met allele was 48.1% in the controls, which is close to other reports in Chinese population [24], but different from other populations, such as Caucasian subjects [22]. Therefore, the differences in the *BDNF* Val66Met genotype distribution frequencies in different ethnicities may result in inconsistency across the studies from the different populations. In addition, it is worth noting that a SNP (rs4074134) near the *BDNF* gene was recently reported to be associated with T2DM independently of obesity in the Chinese Han population [36]. Hence, the role of the *BDNF* polymorphisms and haplotypes in the development of T2DM deserves further examination in different populations.

### BDNF Val66Met variant and cognitive impairments in T2DM patients

The RBANS total score and three index scores- immediate memory, language and delayed memory, were significantly lower in T2DM patients than in healthy subjects. This supports the notion that T2DM has decrements in many aspects of cognitive function [1, 5–7], and increases the risk for some diseases associated with cognitive decline, such as AD and vascular dementia [2–4].

We found that delayed memory index score were significantly lower in the *BDNF*-Met homozygous carriers than in the Val allele carriers in T2DM patients. Further regression analysis found that the number of Met66 alleles was positively correlated with the performance on delayed memory in T2DM patients, supporting that Met-allele may contribute to impaired memory function [9, 12]. However, the exact mechanisms underlying the impact of the Met allele on these cognitive domains are still unclear. Previous reports demonstrated that the *BDNF* Val66Met variant was associated with reductions in hippocampal-dependent memory and in hippocampal volume [12, 13] in control subjects. Moreover, Pezawas *et al.* [37] found that *BDNF* Val66Met polymorphism affected the function of BDNF in neurons, and correlated with several neurological and psychiatric disorders, especially the alteration in human memory. Preclinical studies showed that rats transfected with the *BDNF*-Met allele exhibited a lower secretion of BDNF than those transfected with the Val allele [38] in hippocampal neurons. Accordingly, the *BDNF*-Met variant affects memory and hippocampal function by reducing BDNF transmission within cells and activity-dependent secretion in humans [9]. Furthermore, Met-homozygous carriers show poorer memory function than their Val allele counterparts in humans [9]. In short, these studies indicated that the *BDNF* Met-66 variant may influence memory in humans, with or without T2DM.

### Cognitive deficits and low BDNF serum: association with BDNF Val66Met genotype

The present study demonstrated that lower BDNF levels were positively associated with cognitive performance in T2DM patients, consistent with recent studies demonstrating association of BDNF levels with cognitive function in ageing adults [28]. However, the underlying mechanisms for these results are still unclear. One possible reason is that BDNF has neuroprotective effect, which is involved in regulating synaptic transmission [8] and activity-dependent neuroplasticity that is critical for learning and memory in the hippocampus [9]. It can also induce long-term potentiation (LTP), which is regarded as the neurophysiological mechanisms for learning and memory [10]. Preclinical studies show that hippocampal LTP was markedly reduced in *BDNF* knockout mice, and could be reversed by administrating exogenous BDNF or increasing BDNF expression [9]. Taken together, these findings suggest a close correlation between cognitive function and BDNF level.

However, the origin of peripheral BDNF are still unclear. Although BDNF is mainly expressed in central nervous system, it also occurs in the circulatory system, such as in serum and platelets [39, 40]. Studies have demonstrated that BDNF can pass the blood-brain barrier [39], and serum BDNF levels were found to be associated with that in the brain [40], suggesting that peripheral BDNF may reflect its levels in the brain. Hence, our finding of decreased BDNF may reflect low BDNF brain levels, which could impact cognitive performance in T2DM patients. Indeed, we found that BDNF levels were positively related with delayed memory score in T2DM patients with Met/Met homozygote, while BDNF levels were negatively correlated with the RBANS total and language scores in those with Val/Val homozygote. However, the clinical significance of these discrepant BDNF-cognitive function associations in different *BDNF* genotypes in T2DM patients are still unknown.

Like other studies, we found decreased BDNF levels in T2DM patients [23, 24], which were not associated with the *BDNF* Val66Met genotype in both patients and healthy subjects. However, a recent study found that the Val66Met genotype affected serum BDNF levels, showing that Met-allele carriers had higher levels of BDNF in European populations [41]. Hence the *BDNF* Val66Met polymorphism may correlate with BDNF serum levels in some western population, but not in Asian population. More specifically, a previous study reported that the Met variant was associated with decrease only in the activity-dependent BDNF secretion [17]. The relationship between peripheral BDNF and BDNF gene polymorphisms warrants further investigation.

However, there were several limitations in this study. First, previous studies reported that exercise [42], smoking [43] and alcohol [44] were correlated with cognition. However, unfortunately, we did not collect the data for exercise level, smoking or alcohol drinking in our current study, which will be remedied in future investigation. Second, that Val66Met polymorphism may be related to delayed memory is actually a very important observation in our current study. We should provide another way to double confirm this important finding, such as using MRI test, since some previous studies have shown that Met allele affect human memory-related hippocampal activity in healthy persons [15]. Also Met homozygotes showed decreased gray matter volume [18–20] and worse integrities of fiber tracts in white matter compared to Val allele carriers [21]. Unfortunately, we did not carry out the MRI test in our current study. However, we are performing the MRI test in our ongoing project. Third, our T2DM patients had old age of average 54.9 years and longer duration of illness of average 6 years and had been on oral hypoglycemic, which limit the generalization of our findings to other studies. Fourth, BDNF levels were measured in serum, but not in central nervous system. It remains unknown whether there is a parallel alteration between peripheral BDNF and the central nervous system.

In summary, this study has provided new evidence to support decreased BDNF serum level in T2DM patients, which was significantly associated with the degree of cognitive impairments in T2DM, suggesting that circulating BDNF level may be considered as biomarker of cognitive function in T2DM patients. Also, we found the *BDNF*-Met allele carrier exhibited a poorer delayed memory index score than their Val counterparts in T2DM patients, suggesting that the *BDNF* Val66Met polymorphism was involved in some domains of cognitive deficits in T2DM patients but not in healthy subjects. Furthermore, the association of serum BDNF level with cognitive impairment in T2DM patients was moderated by the *BDNF-*Val66Met polymorphism. Our study, however, is limited by its moderate sample size, wide age range from 20–70 years and longer duration of illness. Additionally, it should be mentioned that that there are significant differences in allele frequency of *BDNF* Val66Met varies between Asian and Caucasian populations, showing that the frequency of Met allele was 48.1% in our present study, which is almost 2.5 times more than that in Caucasian subjects (around 20%) [38]. Thus, it is likely that the specific role of the *BDNF* Met allele in cognitive deficits, especially delayed memory in T2DM patients may be limited to Chinese or Asian populations, which may not be adapted to the western patients. Hence, the association between cognitive impairment and the *BDNF* Met variant in T2DM will need to be confirmed in the future studies in different ethnicities, for instance, in Caucasian population.

## MATERIALS AND METHODS

### Subjects

Three hundred and eleven outpatients (male/female = 136/175) were recruited from the Tangshan Gongren Hospital in TangShan city, 50 miles from Beijing in the period from March 2008 to March 2010. Inclusion criteria included: (1) aged between 20–70 years, Han Chinese; (2) meeting the diagnosis of T2DM according to the World Health Organization 1999 criteria [45]; (3) illness duration < 10 years; (4) without any history of diagnosed coronary artery disease, cerebrovascular disease, stroke, known central nervous system or neuropsychiatric diseases, and any other complications (nephropathy or retinopathy) of diabetes [46, 47]; (5) without any audiovisual or motor coordination impairment affecting the cognitive function tests; (6) be able to complete neurocognitive test. In addition, diabetic nephropathy was defined by increased urinary albumin excretion (UAE) in the absence of other renal diseases, with UAE >20 μg/min [48]. Diabetic retinopathy was defined as the presence of typical retinal microvascular lesions in an individual with diabetes. High-quality fundus photographs of both eyes from all patients were taken and interpreted by a retinal specialist using the International Clinical Diabetic Retinopathy and Diabetic Macular Edema Disease Severity Scales [49]. Diabetic retinopathy was deemed to be present if characteristic lesions were detected (i.e. microaneurisms, hemorrhages, cotton wool spots, intraretinal microvascular abnormalities, hard exudates, and new retinal vessels). All of the patients were receiving conventional medical treatment with the most commonly prescribed drugs being oral hypoglycemics such as Metformin and Repaglinide.

Three hundred and forty-six normal controls (male/female = 138/208) were recruited from the community in Beijing at the same period in parallel with T2DM patients, and matched for sex, age, and education. All controls were in good physical and mental health.

We obtained a complete medical history and conducted physical examinations and laboratory tests for both patients and control subjects. Subjects with any other illnesses, or drug or alcohol abuse/dependence were excluded from this study. All subjects were Han Chinese. They gave signed and informed consent to participate in the study, which was approved by the Institutional Review Board of Tangshan Gongren Hospital.

### Clinical measures

General information, socio-demographic characteristics, medical and psychological conditions of all subjects were collected by a member of the research staff. Additional information was collected from available medical records.

### Cognitive assessment

We individually measured cognitive functioning of all subjects using the Repeatable Battery for the Assessment of Neuropsychological Status (RBANS, Form A) [50]. The RBANS was previously translated into Chinese by our group and its clinical validity and test-retest reliability established among controls and schizophrenia patients [51]. The subjects were administered with the RBANS test on the same day as the blood sample withdrawal. All the subjects performed the cognitive test at least 1 h after breakfast or lunch. No one had taken the cognitive test after an overnight fast.

Four researchers rated subjects on this scale after simultaneously attending a training session in using the RBANS. After training, repeated assessment indicated that the four raters maintained a correlation coefficient for the RBANS total score greater than 0.8. All the four researchers were blinded to all subjects.

### Blood sampling and serum BDNF measurements

We collected serum samples at the same period from patients and normal controls between 7 and 9 a.m. following an overnight fast. The serum was separated, aliquoted, and stored at −70°C before use.

We applied a commercially available, sandwich enzyme linked immunosorbent assay to measure serum BDNF levels, as previously described [52]. All samples were assayed by a research assistant blind to the clinical status. Inter- and intra-assay variation coefficients were 8 and 5%, respectively.

### Genotyping

The genotypes of the *BDNF* Val66Met polymorphism were identified as reported previously [53]. A research assistant who was blind to the clinical status genotyped every subject twice to ensure accuracy.

### Statistical analysis

Deviations from the Hardy–Weinberg equilibrium (HWE) were assessed using the x^2^ goodness-of-fit test. Chi-squared tests were used to compare *BDNF* Val66Met allele and genotype frequencies between T2DM patients and healthy controls. Demographic and clinical variables of the T2DM patients and healthy controls were compared using analysis of variance (ANOVA) for the continuous variables, and chi-squared for the categorical variables. Since the BDNF variables were normally distributed in the patients and normal controls (Kolmogorov-mirnov one-sample test; both *p* > 0.05), the principal outcome analysis consisted of one-way ANOVA. When ANOVA was significant, the effect of sex, age, education, body mass index (BMI), and the clinical variables were tested by adding these variables to the analysis model as covariates. Relationships between variables were assessed with Pearson’s product moment correlation coefficients.

For the main models, the *BDNF* genotype and diagnosis (cases vs controls) were entered as fixed effects. Scores for each cognitive domain and the total scores of RBANS were entered as the dependent variables, with sex, age, and education included as covariates as appropriate. In each model, the main effect of diagnostic group, the main effect of genotype, and diagnostic group × genotype interaction were tested. The diagnostic group × genotype interaction term in the model detects the differential effects that alleles might have on cognitive scores between diagnostic groups. Similarly, the main effect of the *BDNF* genotype on serum BDNF levels was also analyzed using ANCOVA. Bonferroni corrections were applied to each test to adjust for multiple testing.

Lastly, we performed exploratory regression analyses to examine whether the relationships between BDNF serum levels and cognitive function were different across *BDNF* genotype groups. Stepwise multiple regression analysis used RBANS total or Index scores as dependent variables, with BDNF levels as the independent variable in each *BDBF* genotype group. Covariates in these stepwise forward entry models included age, sex, education, BMI, serum total cholesterol (TC) and triacylglycerol (TG), fasting glucose in both the patient and control groups, and clinical variables in the patient group, such as duration of illness and hemoglobin A1c (HbA_1_c). SPSS version 16.0 was used for all of the statistical analyses. Statistical significance was defined as *p* < 0.05.
